# Drug-Diagnostics Co-Development in Oncology

**DOI:** 10.3389/fonc.2014.00208

**Published:** 2014-08-04

**Authors:** Jan Trøst Jørgensen

**Affiliations:** ^1^Dx-Rx Institute, Fredensborg, Denmark

**Keywords:** drug-diagnostic co-development, oncology, companion diagnostics, IHC, FISH, NGS, personalized medicine, precision medicine

The idea of combining drugs and diagnostics in oncology is not new. When the selective estrogen-receptor modulator tamoxifen was developed in the 1970s for the treatment of breast cancer, data on estrogen-receptor status were correlated with the treatment outcome. Based on a phase II study performed in patients with advanced breast cancer, published in 1976, the investigators concluded: “a high degree of correlation between response and positive estrogen-receptor assay suggests the value of the diagnostic test as a means to select patients for tamoxifen treatment” (1). Despite the fact that this conclusion was drawn nearly 40 years ago, the adaptation of the drug-diagnostic co-development model has been relatively slow and it is only within the last decade that it has gained widespread acceptance. The parallel development of the monoclonal antibody trastuzumab (Herceptin^®^, Roche/Genentech) and the companion diagnostics (CDx) assay for HER2 protein overexpression (HercepTest^™^, Dako) in the 1990s seems to have served as an inspiration to the pharma and biotech companies (2, 3), and the number of drug-diagnostic co-development projects within oncology has increased rapidly within the last decade.

Genomic sequencing has shown that marked heterogeneity exists in cancer, both between and within patients, which mean that “standard” treatments seldom work for everyone (4). The taxonomy of classifying the cancer diseases, according to their sites of origin and histology, also seems to be far from optimal when it comes to the treatment decision. The philosophy of “one-disease-one-target-one drug” is history and the improvement in cancer pharmacotherapy must come from an increased understanding of the underlying molecular mechanisms in the individual patient. These mechanisms are of a complex nature and we are far from a complete understanding. However, what we do understand is that drugs work at the molecular level, and it is here that we must seek the solution to a more rational drug development process and the subsequent treatment of the patients in the clinic (5). Molecular diagnostic testing has provided us with an increased understanding of the cancer biology, which has recently enabled the development of molecular-based targeted therapies such as vemurafenib (Zelboraf^®^, Roche/Genentech) for melanoma patients harboring a *BRAF* V600E mutation (6), and crizotinib (Xalkori^®^, Pfizer) and ceritinib (Zykadia^®^, Novartis), for non-small cell lung cancer (NSCLC) patients with *EML4*–*ALK* translocation (7, 8). For the latter two compounds, crizotinib and ceritinib, the development time has been remarkably short, which would never have happened without an in-depth molecular understanding of the disease biology and the mechanism of action of the drugs.

The present research topic of *Frontiers in Oncology* aims to provide an update on the wide-ranging area of drug-diagnostic co-development, biomarker research, and CDx. The research topic covers both basic scientific aspects as well as the clinical and regulatory challenges through a number of Review, Original Research, and Clinical Case Study articles. In the review by Olsen and Jørgensen, an introduction to the subject is given and here both the drug-diagnostic co-development model as well as the clinical and regulatory challenges related to CDx development is discussed (9).

The first CDx to obtain approval by the US Food and Drug Administration (FDA) was the assay for HER2 overexpression (HercepTest^™^, Dako) based on immunohistochemistry (IHC). IHC is a frequently applied method for protein expression analysis in tumor tissue, and despite the current great focus on gene-based assays, especially next-generation sequencing (NGS), this method is still recognized as an important supplement to analysis of different type of gene aberrations. Likewise, there seems to be cancer-related changes in the proteins that are not directly reflected in the changes in RNA and DNA. Gremel et al. review the currently applied CDx tests based on IHC but points also toward the future with regard to mutation-specific antibodies, *in situ* proximity legation assays, and alternative protein binders such as aptamers (10).

Several articles in this research topic touch upon NGS in relation to CDx, but Pant et al. provide the most comprehensive review (11). In this review, the authors exhaustively discuss the different platforms, sequencing technologies, bioinformatics, data reporting, regulatory aspects as well as the potential use of the technology in relation to drug-diagnostic co-development. There is very little doubt that, in the future, NGS will play a prominent role in the development of molecular-based targeted cancer drugs, however, there is still a number of technical, clinical, and regulatory challenges that needs to be overcome.

The review article by Nicolaides et al. suggests a different approach to drug-diagnostic co-development (12). Here, they discuss the use of co-developing diagnostic-targeting vectors to identify patients whose malignant tissue can specifically take up a targeted anti-cancer drug vector prior to treatment. Using this system, the patients can be predetermined in real-time as to whether or not their tumors can specifically take up a drug-linked diagnostic vector, thus inferring the uptake of a similar vector linked to an anti-cancer agent. According to the authors, this approach offers complementary opportunities to the rapid development of broad tumor-specific agents for use in personalized cancer medicine.

Biomarkers may not only serve as an important tool in relation to development of new molecular-based targeted cancer drugs through the drug-diagnostic co-development model but also for repurposing of existing chemotherapeutic anti-cancer drug. The review article by Stenvang et al. describes a strategy of biomarker-guided repurposing of chemotherapeutic drugs for cancer therapy with a specific focus on the topoisomerase I inhibitors and the use of Top1 as a potential predictive biomarker (13).

The recognition of heterogeneity of cancer diseases has called for a rethinking of the clinical trial designs used to demonstrate safety and efficacy of new targeted anti-cancer drugs. The efficacy of these drugs depends on a specific molecular aberration of the tumor that the drug-diagnostics co-development model tries to encounter. In the review by Simon, different clinical trial designs for the parallel development of drugs and diagnostics are discussed both with respect to the use of a single biomarker as well as a genome-wide discovery of a predictive classifier (14).

The development of crizotinib for treatment NSCLC patients with *ALK* rearrangement is definitively a landmark in relation to drug-diagnostic co-development in oncology. This *ALK* rearrangement was discovered in 2007 and already in 2011 crizotinib obtained US FDA approval together with the FISH assay for detection of this specific rearrangement (Vysis *ALK* Break Apart FISH Probe Kit, Abbott Molecular). In the Review/Opinion by Ou et al., the authors discuss the issue of whether the requirements by the US FDA for the simultaneous co-develop of a CDx will delay the approval of receptor tyrosine kinase (RTK) inhibitors for RTK-rearranged NSCLC (15).

Despite great progress in the treatment of cancer achieved with the use of molecular targeted therapy resistance seems to develop to virtually all of the drugs at some point in time. One way to suppress or delay development of resistance might be through the use of combination therapy. In the review article by Goltsov et al., a rational approach to a systematic development of combination therapies is suggested (16). Based on a joint systems analysis of cellular signaling network response and its sensitivity to drug action and oncogenic mutations, they describe an *in silico* method to analyze the targets of drug combinations.

Resistance is also the issue in the research article by Nielsen et al. where the authors look into the link between miR-21 expression and/or cellular localization and resistance to trastuzumab in HER2 positive patients with breast cancer (17). Tumors from 16 HER2 positive patients who underwent adjuvant treatment with trastuzumab were analyzed. Eight of these patients were considered resistant to the treatment. The result of this small study did not show a link between elevated miR-21 expression and resistance to adjuvant treatment with trastuzumab. However, more studies will be needed in order to prove or eliminate the role of miR-21.

In a clinical case study article by Russell et al., tumor profiling for two patients has been described (18). Both patients had advanced-stage cancer and failed standard treatment. The article describes how tumor profiling was used together with a systematic literature review (Caris Molecular Intelligence™) that was used to identify potential beneficial treatments for the patients resulting in disease remission in both cases.

The use of molecular diagnostics has given us new insight into the cancer disease biology, which has enabled development of new anti-cancer drugs with much more specific and well-defined mechanisms of action. When this knowledge is translated into the drug-diagnostic co-development model, remarkable results can be achieved. Crizotinib is one such example, and a similar or even more remarkable example is the recent development of ceritinib, another ALK inhibitor for NSCLC patients with *ALK* rearrangement. In the spring of 2014, ceritinib obtained an accelerated FDA approval based on efficacy data from only 163 metastatic NSCLC patients enrolled in a phase I single-arm, open-label clinical trial (19). Such a result is only achievable with the use of a CDx that enables pre-selection of the patients who are likely responders to the drug, which as for ceritinib resulted in a response rate above 50% even in a phase 1 trial. Despite the challenges that anti-cancer drug development faces, especially the development of resistance to the molecular targeted drugs, the drug-diagnostic co-development model has shown to be an invaluable tool in oncology, which definitively point to the future.

## Conflict of Interest Statement

Jan Trøst Jørgensen is working as a consultant for Dako and Euro Diagnostica and has given lectures at meetings sponsored by Roche and AstraZeneca.
